# Transpapillary biliary drainage using a forward-viewing endoscope for patients with distal malignant biliary obstruction and type I duodenal stenosis

**DOI:** 10.1055/a-2554-2784

**Published:** 2025-04-04

**Authors:** Yuichi Hirata, Kazuhiro Iida, Kei Takahashi, Mariko Hatada, Kana Miyara, Yuichiro Aoyama, Ryosuke Mizukami, Takahiro Oribe, Mizuka Yonezawa, Daisuke Orita, Ryutaro Yoshida, Michitaka Kouhashi, Takuya Mimura, Akihiko Nishizawa, Yoshihide Ueda, Kenzo Yamashiro, Yoshihiro Okabe

**Affiliations:** 1 469536 Gastroenterology, Kakogawa Central City Hospital, Kakogawa, Japan

**Keywords:** Pancreatobiliary (ERCP/PTCD), Strictures, ERC topics

## Abstract

**Background and study aims:**

Distal malignant biliary obstruction and duodenal stenosis may be complicated in patients with pancreaticobiliary cancer. It is often difficult to insert a side-viewing duodenoscope and perform transpapillary biliary drainage in patients with duodenal stenosis on the oral side of the major papilla; hence, in this study, we attempted transpapillary biliary drainage using a forward-viewing endoscope and reported its efficacy and safety.

**Patients and methods:**

This retrospective single-center cohort study included 12 patients (17 sessions) who underwent transpapillary biliary drainage using a forward-viewing endoscope between April 2020 and October 2024. The tip of the forward-viewing endoscope was inverted around the inferior duodenal angulus and the major papilla was viewed from the anal side. We evaluated patient characteristics, outcomes, and adverse events (AEs) during these procedures.

**Results:**

Biliary cannulation and drainage were successful in all cases, with a median cannulation and procedure time of 7 minutes (range 0.5–34) and 33 minutes (range 10–101), respectively. Median biliary cannulation time required was 3.5 minutes (range 0.5–15) for 10 sessions in patients with a history of endoscopic sphincterotomy and 9 minutes (range 4–34) for seven sessions in patients with native papilla ( *P* = 0.01). The types of biliary drainage were plastic stent in nine sessions, endoscopic nasobiliary drainage in two sessions, and self-expandable metal stent in six sessions. Hyperamylasemia as AEs occurred in three sessions.

**Conclusions:**

Transpapillary biliary drainage using a forward-viewing endoscope is a useful option for patients with type I duodenal stenosis.

## Introduction

Endoscopic retrograde cholangiopancreatography (ERCP) has been developed as a diagnostic method for biliary and pancreatic diseases. Duodenoscopy using a fiberscope was first reported in 1958 1 , whereas pancreatography using a duodenoscope was first reported in 1968 2 . Subsequently, Takagi K et al. reported cholangiography for the first time 3 . Recently, the indication for ERCP has changed from diagnosis to treatment, owing to advances in minimally invasive examinations such as computed tomography (CT), magnetic resonance cholangiopancreatography, and endoscopic ultrasound (EUS). Today, transpapillary biliary drainage using a side-viewing duodenoscope is widely performed in patients with obstructive jaundice, which is caused by various diseases such as pancreatic cancer, common bile duct stones, and cholangiocarcinoma. In patients with pancreaticobiliary cancer, distal malignant biliary obstruction and duodenal stenosis may be complicated, and are classified as type I to III according to the anatomical location of the duodenal stenosis in relation to the major papilla 4 5 . Type I stenosis occurs on the oral side of the major papilla but does not involve the major papilla; type II stenosis affects the second part of the duodenum with involvement of the major papilla; and type III stenosis involves the third part of the duodenum, without involvement of the major papilla. It is often difficult to insert a side-viewing duodenoscope, particularly in patients with type I duodenal stenosis; hence, EUS-guided biliary drainage (EUS-BD) is a useful alternative procedure. However, this procedure is not yet widely performed because it requires advanced techniques. This study aimed to report the efficacy and safety of transpapillary biliary drainage using a forward-viewing endoscope.

## Patients and methods

The study protocol was approved by the Institutional Review Board of the Kakogawa Central City Hospital (IRB No. 2022–04). The study was conducted in accordance with the Declaration of Helsinki as revised in Fortalenza, Brazil, in 2013. All authors had full access to the study data and accepted responsibility for submission for publication.

### Patients

This was a single-center, retrospective study conducted at Kakogawa Central City Hospital. We investigated clinical data from 2781 consecutive patients who underwent ERCP between April 2020 and October 2024. Routine medical history inquiries, physical examinations, imaging evaluations, and blood tests were performed in all patients. This study included 12 patients (17 sessions) who underwent transpapillary biliary drainage using a forward-viewing endoscope because of the difficulty in inserting a side-viewing duodenoscope due to type I duodenal stenosis. Written informed consent was obtained from each patient before the procedure.

### Procedures

**Fig. 1:**
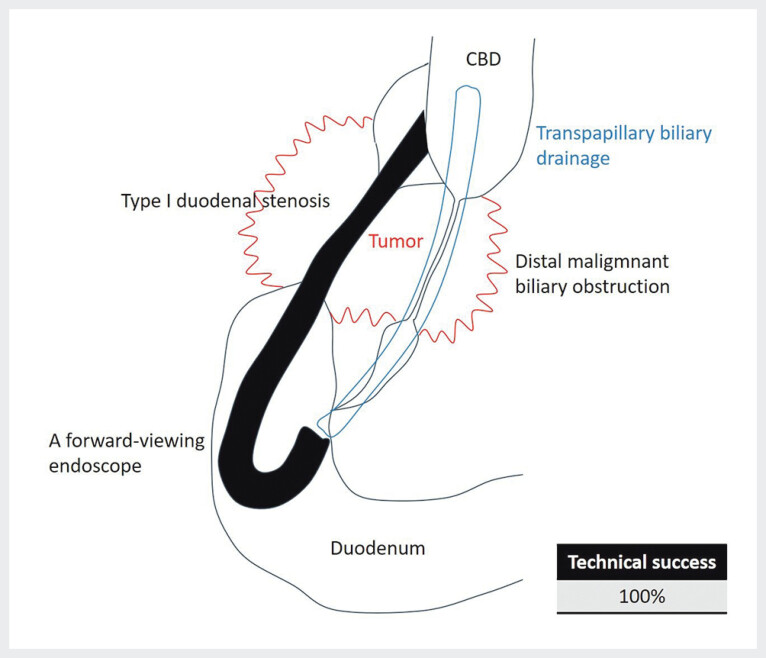
Graphical image. Transpapillary biliary drainage using a forward-viewing endoscope is a useful option for patients with distal malignant biliary obstruction and type I duodenal stenosis.

Procedures were performed by endoscopists with experience performing 300 or more procedures annually for at least 5 years. The procedures were performed under conscious sedation using a combination of intravenous flunitrazepam and pentazocine. Patients were placed in the prone position, administered oxygen, and oxygen saturation and electrocardiogram monitoring were performed. A side-viewing duodenoscope (JF-260V or TJF-260V or TJF-Q290V; Olympus Medical Systems, Tokyo, Japan) was inserted into the duodenal bulb, but it was not possible to pass through type I duodenal stenosis. Therefore, the balloon anchor technique was performed; however, it was difficult to insert the scope into the second part of the duodenum. After passing through the duodenal stenosis using a forward-viewing endoscope (SIF-H290S or PCF-PQ260L or PCF-H290TI; Olympus Medical Systems, Tokyo, Japan) with a clear hood attached to the tip, the tip of the scope was carefully inverted around the inferior duodenal angulus, and the major papilla was observed from the anal side. Biliary cannulation was performed using a conventional ERCP catheter (MTW ERCP catheter; ABIS, Tokyo, Japan) and 0.025-inch guidewire (VisiGlide2; Olympus Medical Systems, Tokyo, Japan or EndoSelector; Boston Scientific Japan, Tokyo, Japan). In general, bile duct cannulation is performed using contrast- or wire-guided cannulation. The types of endoscopic transpapillary biliary drainage involved endoscopic biliary stenting (EBS) and endoscopic nasobiliary drainage (ENBD). We placed a plastic stent (PS) or self-expandable metal stent (SEMS) for EBS, or a nasodrainage catheter for ENBD ( Fig. 1 ).

### Outcome definition

We retrospectively examined the following parameters: patient characteristics; details of endoscopic procedures; outcomes, including technical and clinical success; and adverse events (AEs).

The primary outcome was technical success, which was defined as successful deployment of transpapillary biliary drainage in the marked location with sufficient coverage of the biliary stricture. Secondary outcomes were evaluation of patient characteristics, clinical success, and AEs. Outcomes and AEs were evaluated according to TOKYO criteria 2014 6 . Clinical success was defined as a 50% decrease in or normalization of bilirubin level within 14 days of stent placement 6 . Total procedure time was defined as time for the procedure using a forward-viewing endoscope, excluding time for side-viewing duodenoscope insertion and the balloon anchor technique.

### Statistical analysis

Biliary cannulation time, defined as reaching the papilla to completion of biliary cannulation, and total procedure time were statistically analyzed in 10 patients with a history of endoscopic sphincterotomy (EST) and seven patients with native papilla using the Mann-Whitney’s U test. Statistical significance was set at *P* < 0.05. All analyses were performed using SPSS Statistics version 26 (IBM, Armonk, New York, United States).

## Results

### Patient characteristics

Characteristics of all the patients who underwent transpapillary biliary drainage using a forward-viewing endoscope during the study period are shown in Table 1 . Median age was 66 years (range 51–88) and there were six women (50.0%) included in the study. Primary diseases were pancreatic (n = 9) and biliary tract cancer (n = 3), which were pathologically diagnosed as adenocarcinoma. These patients were considered unsuitable for surgery because of advanced tumor stage. Tumors were localized in the pancreatic head (n = 7 cases), groove region (n = 1 case), pancreatic body (n = 1 case), intrahepatic bile duct (n = 1 case), distal bile duct (n = 1 case), and gallbladder (n = 1 case). In cases in which the location of the cancer was the intrahepatic bile duct and gallbladder, the tumors had grown very large and directly invaded the superior duodenal angulus (SDA) and distal bile duct. In a case in which the location of the cancer was the distal bile duct, the tumor progressed toward the hilar bile duct and invaded the SDA. There were seven patients (n = 10 sessions) with a history of EST and five patients (n = 7 sessions) with native papilla. Nine sessions were concomitant with cholangitis, including grade I (mild) in three sessions, grade II (moderate) in five sessions, and grade III (severe) in one session, according to the Tokyo Guidelines 2018 7 .

**Table:** **Table 1** Patient characteristics.

		Case	Session
N		12	17
Age, years, median (range)		66 (51–88)	
Female gender, n (%)		6 (50.0)	8 (47.1)
Disease, n (%)	Pancreatic cancer	9 (75.0)	14 (82.3)
	Biliary tract cancer	3 (25.0)	3 (17.7)
Histology/cytology, n (%)	Adenocarcinoma	12 (100)	17 (100)
Tumor location, n (%)	Pancreatic head	7 (58.3)	12 (70.6)
	Groove region	1 (8.3)	1 (5.9)
	Pancreatic body	1 (8.3)	1 (5.9)
	Intrahepatic bile duct	1 (8.3)	1 (5.9)
	Distal bile duct	1 (8.3)	1 (5.9)
	Gallbladder	1 (8.3)	1 (5.9)
Stage, n (%)	III	6 (50.0)	8 (47.1)
	IV	6 (50.0)	9 (52.9)
Resectability classification, n (%)	Pancreatic cancer		
	BR-A	2 (16.7)	3 (17.6)
	UR-LA	4 (33.3)	5 (29.4)
	UR-M	3 (25.0)	6 (52.9)
	Biliary tract cancer		
	Unresectable	3 (25.0)	3 (17.6)
Ampullary interventions, n (%)	Native papilla	5 (41.7)	7 (41.2)
	History of EST	7 (58.3)	10 (58.8)
Concomitant with cholangitis, n (%)	Nothing	6 (50.0)	8 (47.1)
	Grade I (mild)	3 (25.0)	3 (17.7)
	Grade II (moderate)	2 (16.7)	5 (29.4)
	Grade III (severe)	1 (8.3)	1 (5.9)
NOTE: Tumor staging was based on guidelines from the 8th edition of the Union for International Cancer Control (UICC) TNM classification. Resectability classification was based on General Rules for the Study of Pancreatic Cancer, Japan Pancreas Society 8thedition. Severity grading of cholangitis was based on Tokyo Guidelines 2018. BR-A, borderline resectable with arterial involvement; EST, endoscopic sphincterotomy; UR-LA, unresectable stage with local advanced factors; UR-M, unresectable stage with metastatic factors.			

### Forward-viewing endoscopes

Endoscope specifications are listed in Table 2 . The bending angle of the forward-viewing endoscopes is larger than that of the side-viewing duodenoscopes. The outer diameter of the distal end and insertion tube of the forward-viewing endoscopes were smaller than those of the side-viewing duodenoscopes. Therefore, the working length of the forward-viewing endoscopes was longer than that of the side-viewing duodenoscopes, and the channel inner diameter of the forward-viewing endoscopes was narrower than that of the side-viewing duodenoscopes.

**Table:** **Table 2** Specifications for endoscopes used in this study.

**Forward-viewing endoscope**	**Viewing angle**	** Bending angle (up, down, right, left) **	**Distal end outer diameter**	**Insertion tube outer diameter**	**Working length**	**Channel diameter**
SIF-H290S	140 ^°^	180 ^°^ , 180 ^°^ , 160 ^°^ , 160 ^°^	9.2 mm	9.2 mm	1520 mm	3.2 mm
PCF-H290TI	140 ^°^	210 ^°^ , 180 ^°^ , 160 ^°^ , 160 ^°^	9.8 mm	10.5 mm	1330 mm	3.2 mm
PCF-PQ260L	140 ^°^	180 ^°^ , 180 ^°^ , 160 ^°^ , 160 ^°^	9.2 mm	9.2 mm	1680 mm	2.8 mm
**Side-viewing duodenoscope**	**Viewing angle**	** Bending angle (up, down, right, left) **	**Distal end outer diameter**	**Insertion tube outer diameter**	**Working length**	**Channel diameter**
JF-260V	100 ^°^	120 ^°^ , 90 ^°^ , 110 ^°^ , 90 ^°^	12.6 mm	11.3 mm	1240 mm	3.7 mm
TJF-260V	100 ^°^	120 ^°^ , 90 ^°^ , 110 ^°^ , 90 ^°^	13.5 mm	11.3 mm	1240 mm	4.2 mm
TJF-Q290V	100 ^°^	120 ^°^ , 90 ^°^ , 110 ^°^ , 90 ^°^	13.5 mm	11.3 mm	1240 mm	4.2 mm

### Outcomes

**Fig. 2:**
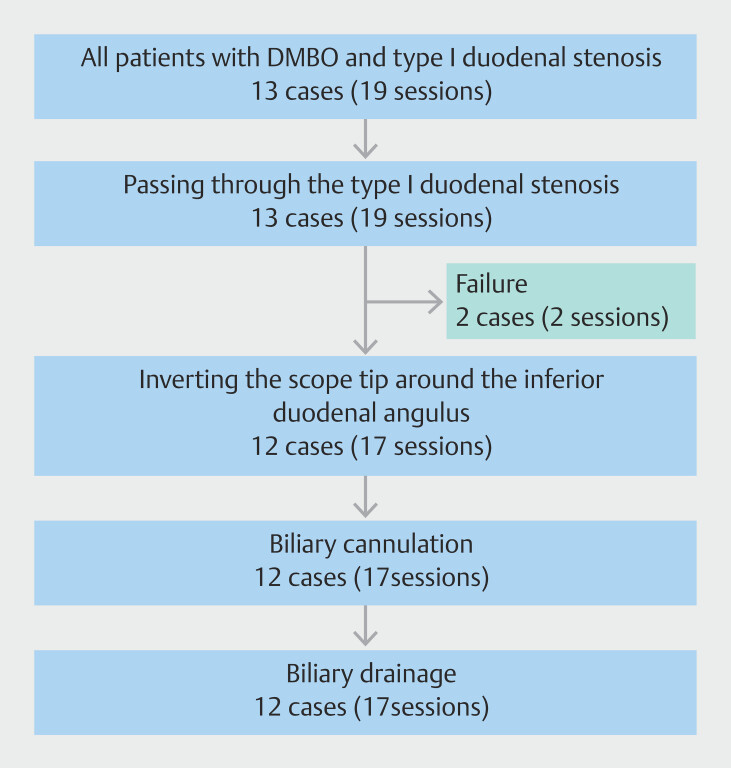
Flowchart of this procedure.

The flowchart of this procedure is shown in Fig. 2 . In all 13 cases (n = 19 sessions) with distal malignant biliary obstruction and type I duodenal stenosis, it was possible to pass through the duodenal stenosis. However, in two cases, it was difficult to invert the tip of a forward-viewing endoscope in the duodenum. In one case, it was difficult from the first attempt, and in the other case, this procedure was successful in the first session, but the duodenal lumen narrowed due to tumor progression, making inversion difficult in the second session. In this study, we focused on 12 cases (n = 17 sessions) in which the tip of a forward-viewing endoscope was successfully inverted.

Outcomes of this study are summarized in Table 3 . Biliary cannulation and drainage were successful in all sessions, with a technical success rate of 100% (17/17).

**Table:** **Table 3** Outcomes of transpapillary biliary drainage using a forward-viewing endoscope.

	Session	Ampullary intervention		
		History of EST	Native papilla	*P* value
N	17	10	7	
Technical success, n (%)	17 (100)	10 (100)	7 (100)	–
Clinical success, n (%)	16 (94.1)	9 (90.0)	7 (100)	0.58
Cannulation time, median (range)	7 (0.5–34)	3.5 (0.5–15)	9 (4–34)	0.01
Total procedure time, median (range)	33 (10–101)	28 (10–101)	35 (21–88)	0.49
Forward-viewing endoscope, n (%)				
SIF-H209S	11 (64.7)	6 (60.0)	5 (71.4)	
PCF-H290TI	5 (29.4)	3 (30.0)	2 (28.6)	
PCF-H290TI alone	2 (11.8)	1 (10.0)	1 (14.3)	
After procedure failed with SIF-H290S	3 (17.6)	2 (20.0)	1 (14.3)	
PCF-PQ260L	1 (5.9)	1 (10.0)	0 (0.0)	
Biliary cannulation, n (%)				
Contrast-assisted cannulation	6 (35.3)	6 (60.0)	0 (0.0)	
Wire-guided cannulation	10 (58.8)	4 (40.0)	6 (85.7)	
Pancreatic guidewire cannulation	1 (5.9)	0 (0.0)	1 (14.3)	
Biliary drainage, n (%)				
EBS (PS)	9 (52.9)	4 (40.0)	5 (71.4)	
ENBD	2 (11.8)	2 (20.0)	0 (0.0)	
EBS (SEMS)	6 (35.3)	4 (40.0)	2 (28.6)	
Adverse events, n (%)	3 (17.6)	0 (0.0)	3 (42.9)	
Hyperamylasemia, n (%)	3 (17.6)	0 (0.0)	3 (42.9)	
NOTE: Outcomes and adverse events were evaluated according to TOKYO criteria 2014. EBS, endoscopic biliary stenting; ENBD, endoscopic nasobiliary drainage; EST, endoscopic sphincterotomy; SEMS, self-expandable metal stent.				

Clinical success rates were 94.1% (16/17), because cholangitis was not improved in one session in which PS was utilized.

Median time of biliary cannulation and total procedure time were 7 minutes (range 0.5–34) and 33 minutes (range 10–101), respectively.

Procedures were performed in 11 sessions using SIF-H290S, five sessions using PCF-H290TI, and one session using PCF-PQ260L. Three of the five sessions using PCF-H290TI were performed because SIF-H290S was bent and could not pass through the duodenal stenosis.

Types of biliary cannulation were conventional contrast-assisted cannulation in six sessions, wire-guided cannulation in 10 sessions, and pancreatic guidewire cannulation in one session.

**Fig. 3:**
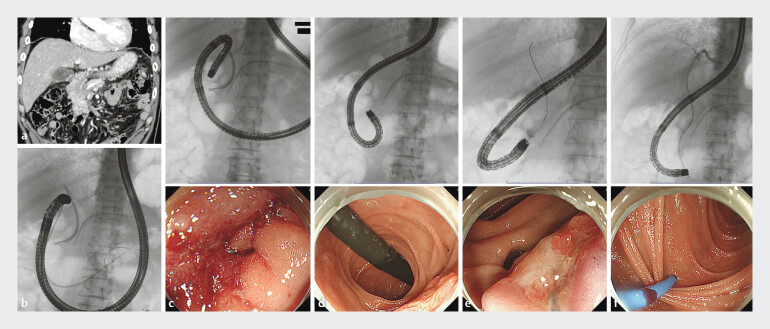
Fluoroscopic images. **a** A 63-year-old man was admitted due to increasing cholangitis and jaundice. Tumor on the pancreatic body had spread to the pancreatic head and invaded the superior duodenal angulus. **b** Because of stenosis of the superior duodenal angulus, a side-viewing duodenoscope could not passed through even using balloon anchor technique. **c** A forward-viewing endoscope (PCF-PQ260L) passed through the duodenal stenosis. **d** The tip of the scope was inverted around the inferior duodenal angulus and the major papilla was observed from the anal side, and EBS was removed. **e** Biliary cannulation was performed using a conventional ERCP catheter and 0.025-inch guidewire. **f** A plastic stent (6F) for EBD was placed.

**Fig. 4:**
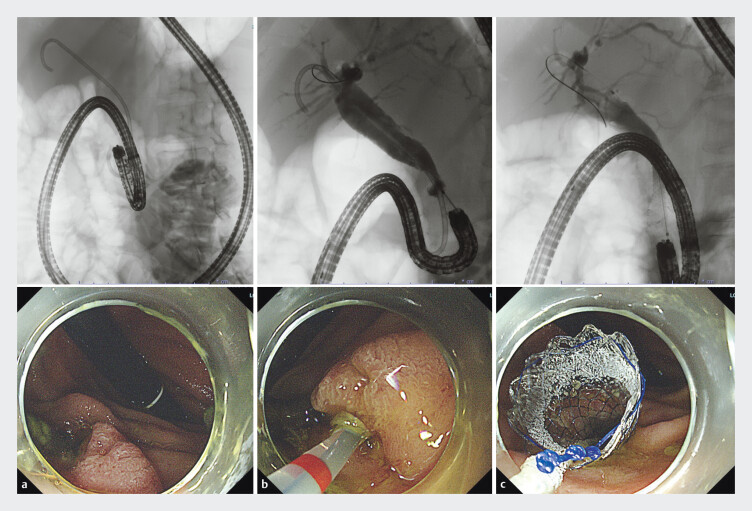
Fluoroscopic images. **a** A 55-year-old woman with pancreatic cancer was admitted for replacement of EBS. A forward-viewing endoscope (SIF-H290S) passed through the duodenal stenosis and the tip of the scope was inverted around the inferior duodenal angulus. **b** Biliary cannulation was performed. **c** A fully-covered SEMS (10 mm × 6 cm) for EBD was placed.

Types of transpapillary biliary drainage were EBS (PS) in nine sessions ( Fig. 3 ), ENBD in two sessions, and SEMS in six sessions ( Fig. 4 ). There were 10 sessions in patients with a history of EST and seven sessions in patients with native papilla. Median biliary cannulation times in patients with a history of EST and in patients with native papilla were 3.5 minutes (range 0.5–15) and 9 minutes (range 4–34), respectively ( *P* = 0.01). In contrast, median total procedure times in patients with a history of EST and in patients with native papilla were 28 minutes (range 10–101) and 35 minutes (range 21–88), respectively ( *P* = 0.49).

### Adverse events

AEs in this study are also shown in Table 3 . Hyperamylasemia as an AE occurred in three sessions (17.6%), all of which were in patients with native papilla. They were asymptomatic and improved with conservative treatment. There were no other AEs, such as bleeding, post-ERCP pancreatitis, or perforation of the gastrointestinal tract or bile duct, in any session.

## Discussion

Transpapillary biliary drainage is often challenging in patients with type I duodenal stenosis. A recent study suggested that scar stenosis formed by duodenal bulb ulcers often makes it difficult for a side-viewing duodenoscope to pass through the stenosis and increases risk of duodenal perforation 8 . EUS-BD is a useful alternative procedure for these patients; however, it requires an advanced technique and is performed mainly at high-volume centers. In general hospitals, it is often difficult to perform EUS-BD immediately for patients requiring emergency drainage because of concomitant acute cholangitis. In addition, percutaneous transhepatic biliary drainage (PTBD) and EUS-BD for patients with malignant biliary obstruction theoretically carry a risk of inducing peritoneal dissemination of cancer cells due to bile leakage. Therefore, transpapillary biliary drainage, a physiological drainage route, is more desirable. In this study, transpapillary biliary drainage using a forward-viewing endoscope demonstrated high efficacy and safety in patients with distal malignant biliary obstruction and type I duodenal stenosis. Biliary cannulation was significantly easier in patients with a history of EST than in those with native papilla.

There are some reasons for selecting this procedure. First, interstitial pneumonia in some patients was severe, making it difficult to hold their breath even for a short time; therefore, PTBD could not be performed. Second, EUS-BD was also difficult to perform because of ascites or development of collateral circulation in the lesser curvature of the stomach caused by portal vein obstruction associated with tumor invasion 5 : therefore, transpapillary biliary drainage was more feasible. Among our cases, the reasons for selecting this procedure were difficulty in holding breath in one case (n = 1 session), ascites in three cases (n = 5 sessions), and collateral circulation in six cases (n = 9 sessions).

In many cases, the stomach was prone to bending; hence, we chose a longer endoscope instead of a gastroscope. Forward-viewing endoscopes have better tip mobility and an advantage in passing through the duodenal stenosis owing to the smaller diameter of the endoscope compared with side-viewing duodenoscopes. On the other hand, the longer working length and narrow channel inner diameter of forward-viewing endoscopes were limiting factors in selection of procedure devices. PCF-PQ260L has the longest working length, so the lower end of EBS(PS) integrated with the delivery system could not be pushed out from the tip of the scope, making it difficult to place PS. Therefore, a cut ENBD (6F) was used as the EBS ( Fig. 3 ). Because the PCF-H290TI has a large outer scope diameter and greater stiffness, it was easier to utilize when passing through the stenosis when the SIF-H290S was bent and could not pass through the duodenal stenosis. However, inverting the scope tip of the PCF-H290TI may be difficult unless there is sufficient working space around the inferior duodenal angulus. The optimal scope for this procedure will need to be evaluated based on future accumulation of similar cases, but we believe that SIF-H290S is optimal in terms of working length, outer diameter of the scope, and channel inner diameter. In addition, it was considered that attachment of a clear hood to the tip of the endoscope helped to safely perform inversion in the narrow duodenal lumen and facilitated observation of the papilla from the anal side. Therefore, it is important to confirm prior to the procedure, using CT and fluoroscopy, if there is sufficient working space around the inferior duodenal angulus to allow the scope to be inverted.

Usefulness of ERCP using several forward-viewing endoscopes has been reported, mainly in patients with surgically altered anatomy, such as those undergoing Billroth II, Roux-en-Y or pancreaticoduodenectomy 9 10 . However, there are few reports on patients with normal anatomy. ERCP or direct cholangioscopy using an ultrathin endoscope has also been reported 11 12 13 14 15 . In addition, usefulness of a forward-viewing endoscope for biliary cannulation in patients with periampullary diverticula or intradiverticular papilla has been reported 16 17 18 . Almost all of these previous reports were in benign diseases, such as bile duct stones. To our knowledge, this is the first summary report of transpapillary biliary drainage using a forward-viewing endoscope for malignant stenoses of both the distal bile duct and duodenum on the oral side of the major papilla.

Limitations of this study include its single-center retrospective design and small sample size, which future studies should address. When using a forward-viewing endoscope, malignant tumor around the duodenum invades the duodenum from the outside, it is possible affecting not only the duodenal lumen stricture, but also fixation of adhesions around the duodenum, making it difficult to manipulate the scope even if the tip of the forward-viewing endoscope passes through the stricture, and making it difficult for the scope to reach the anal side of the major papilla located in the duodenum second portion in cases of type I duodenal stricture accompanying pancreaticobiliary cancer. Because this procedure using forward-viewing endoscopes carries a risk of gastrointestinal perforation, it is important that it be performed carefully by an experienced expert endoscopist after confirming that there is sufficient space around the inferior duodenal angulus. No gastrointestinal perforation occurred in our cases.

## Conclusions

In conclusion, transpapillary biliary drainage using a forward-viewing endoscope is a useful option in patients with distal malignant biliary obstruction and type I duodenal stenosis.
